# Incidence and mortality of cervical cancer in Vietnam and Korea (1999-2017)

**DOI:** 10.4178/epih.e2020075

**Published:** 2020-12-16

**Authors:** Kim Ngoc Tran, Yoon Park, Byung-Woo Kim, Jin-Kyoung Oh, Moran Ki

**Affiliations:** Department of Cancer Control and Population Health, Graduate School of Cancer Science and Policy, National Cancer Center, Goyang, Korea

**Keywords:** Cervical cancer, Incidence, Mortality, Vietnam, Korea

## Abstract

**OBJECTIVES:**

Cervical cancer is a major disease burden in Vietnam. This study aimed to estimate the incidence and mortality rates of cervical cancer in Vietnam (1999-2017) in comparison to those in Korea, where a population-based cancer registry and national cervical cancer screening program have been implemented.

**METHODS:**

The estimated incidence and mortality of cervical cancer in Vietnam and Korea (1999-2017) were collected from Global Burden of Disease 2017 study. Estimated age-standardized rates (ASRs) in both countries were calculated utilizing the 1999-2017 population of each country and the World Health Organization standard population. The reported ASRs in Korea were also computed using data on incidence and mortality (1999-2017) and the Korean population from the Korea Statistical Information Service.

**RESULTS:**

In Vietnam, the estimated incidence and mortality of cervical cancer decreased annually by 0.84% and 1.01%. In Korea, the trend of reported incidence showed a dramatic drop (1999-2007 annual percent change [APC], -4.53%) before stably declining (2007-2017 APC, -2.71%). Reported mortality also significantly decreased (2003-2008 APC, -6.63%), and then maintained a stable decline (2008-2017 APC, -3.78%). The incidence and mortality rates were higher in Vietnam than in Korea. The declining trend of incidence and mortality in Vietnam was slower than the corresponding trends in Korea.

**CONCLUSIONS:**

A national screening program should be implemented for Vietnamese women aged over 30 to maintain, or even hasten, the decline in cervical cancer incidence and mortality. A population-based cancer registry may help monitor the effectiveness of a cervical cancer screening program.

## INTRODUCTION

Cervical cancer is a globally important health issue, as it is the fourth most frequent cancer among women in terms of both incidence (13.1 per 100,000) and mortality (6.9 per 100,000) [1]. In Vietnam, cervical cancer ranks as the seventh leading type of cancer overall among women and the most common gynecologic cancer (7.1 per 100,000) [2,3]. Cervical cancer is predicted to become 1 of the top 5 most frequently diagnosed cancers in women in Vietnam in the next decade [4].

A reduction in the burden of cervical cancer has been observed in several countries that have successfully implemented nationwide screening programs. Korea, for instance, is an Asian country that has organized effective programs for cervical cancer control and prevention. Before 1999, cervical cancer was among the top 5 most commonly diagnosed cancers in Korea. Since the National Cancer Screening Program was initiated in 1999, the age-standardized rates (ASRs) of incidence and mortality for cervical cancer have significantly decreased [5-7]. Nationwide efforts to implement other prevention programs, such as human papilloma virus (HPV) vaccination programs and reproductive education, have also helped to ease the cervical cancer burden in Korea. Furthermore, the Korean cancer registry provides high-quality data for evaluating the effectiveness of these programs.

In Vietnam, a national screening program has not yet been implemented. However, an opportunistic screening program has been organized following the guidelines of the Vietnam Ministry of Health since 2011 [8]. According to the guidelines, it is recommended to provide cervical cancer screening by utilizing cytology and visual inspection with acetic acid (VIA) tests for women aged 21-70 years, with a particular focus on the 30-year to 50-year group [8]. Since screening tests are not included in the National Health Insurance program, individuals have been obligated to pay out of pocket for cervical cancer screening. Therefore, Vietnamese women have few opportunities to access screening tests due to a lack of knowledge and poverty [9].

Vietnam has 63 cities and provinces with over 96 million people, but only 9 regional cancer registries have been established [9]. Among these 9 registries, the cancer registry data from Hanoi and Ho Chi Minh City have been selectively used to estimate the global burden of cancer in databases, such as the Cancer Incidence in Five Continents (CI5) by the International Agency for Research on Cancer (IARC) or the Global Burden of Disease 2017 (GBD 2017) study performed by the Institute for Health Metrics and Evaluation (IHME), after evaluating data quality using relevant indices [10-14].

In light of the circumstances of Vietnam, the aims of this study were to overview the incidence and mortality of cervical cancer among Vietnamese women in comparison with the corresponding rates in Korea, where national cervical cancer screening has been implemented through the comprehensive establishment of a nationwide cancer registry.

## MATERIALS AND METHODS

### Source of data

The estimated number of new cases and deaths in Vietnam and Korea due to cervical cancer (1999-2017) were derived from the GBD 2017. These data were available and collected directly from the GBD Results Tool of IHME (http://ghdx.healthdata.org/gbd-results-tool). Cervical cancer was classified according to the International Classification of Diseases (ICD; using both ICD-9 and ICD-10) for oncology (C53: cervix uteri) [15]. According to the definition of the GBD 2017, the estimated incidence, in terms of cases per year, was defined as the number of new cases diagnosed during a given year in a specified population, and the estimated number of deaths was counted as the number of deaths from a given cause occurring in a specified population per year. These data were incorporated from the published literature, surveillance data, survey data, hospital and clinical data, and other types of data [14,15].

To compare the estimated and the reported data for both the incidence and mortality rates of cervical cancer in Korea, we used data in Korea from 2 separate sources: the estimated incidence and mortality in Korea from the GBD 2017, and the number of new cases and deaths occurring in Korea from the Korean Statistical Information Service (KOSIS) (available from http://www.kosis.kr). Since the available data for incidence and mortality in Korea were limited to the range from 1999 to 2017, we decided to collect the estimated data for cervical cancer incidence and mortality in Vietnam from the GBD 2017 for the same time period (1999-2017).

### Statistical analysis

To calculate the estimated ASRs for incidence and mortality in Vietnam and Korea, age-specific rates were computed for each year using the estimated incident cases and deaths from the GBD 2017 and the distribution of the women population in each country (1999-2017) from the World Population Prospects 2019 Project [16].

To calculate the ASRs for reported incidence and mortality in Korea, reported data were collected and corrected from the KOSIS after being submitted by the Korea Central Cancer Registry (KCCR). The KCCR is a national population-based data registry, and the data quality of the KCCR is annually evaluated by using 3 indices: the proportion of death certificate (DCO%) only, the proportion of microscopic verification (MV%), and the mortality/incidence (M/I) ratio [17]. In 2019, several indices of quality were examined in an annual report published to assess the quality of the data from 2017 [17]. In term of completeness and validity, DCO% was computed as 0.8% and 1.0% for men and women, respectively. MV% for the diagnosis was 89.7% for men and 92.8% for women. The M/I ratio was reported to be 40.4 and 27.5 for men and women [17]. The ASRs of reported incidence and mortality were computed using the distribution of the Korean women population (1999-2017) derived from the KOSIS.

For comparison, we used the World Health Organization (WHO) world standard population to calculate the estimated ASRs in Vietnam and Korea, and the reported ASRs in Korea [18].

The annual percent change (APC), defined as the average percent change of ASRs, was utilized to illustrate trends in cancer rates over a given time [19]. The APCs for the estimated ASR incidence and mortality rates in both countries, as well as the APCs for the reported ASRs of incidence and mortality in Korea, were analyzed using the Joinpoint Regression Analysis program provided by the Surveillance Research program of the US National Cancer Institute version 4.8.0.1 (https://surveillance.cancer.gov/joinpoint/).

### Ethics statement

This study used the secondary data from two available databases GBD 2017 study and the KOSIS, that are not individually identifiable and therefore analysis would not involve human subjects.

## RESULTS

**Figure 1.**
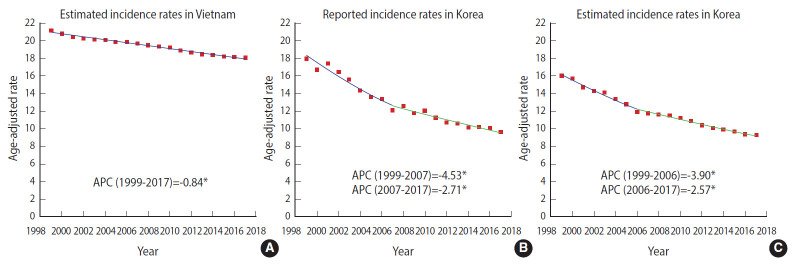
Trends in (A) estimated age-standardized incidence in Vietnam, (B) reported incidence rates in Korea, and (C) estimated age-standardized incidence in Korea for cervical cancer, 1999-2017. Age-adjusted rates: age-standardized rate (ASR) per 100,000 women. ASRs were calculated using the estimates of incidence for cervical cancer in Vietnam and Korea obtained from Global Burden of Disease Study 2017, annual population from World Population Prospects 2019; reported ASRs were calculated using incidence data, and annual population of Korea from Korea Statistical Information Service; and World Health Organizaion new world standard population. ASR and annual percent changes (APCs) were computed by using Joinpoint Regression Analysis program provided by the Surveillance Research Program of the US National Cancer Institute. ^*^p<0.05.

**Figure 2.**
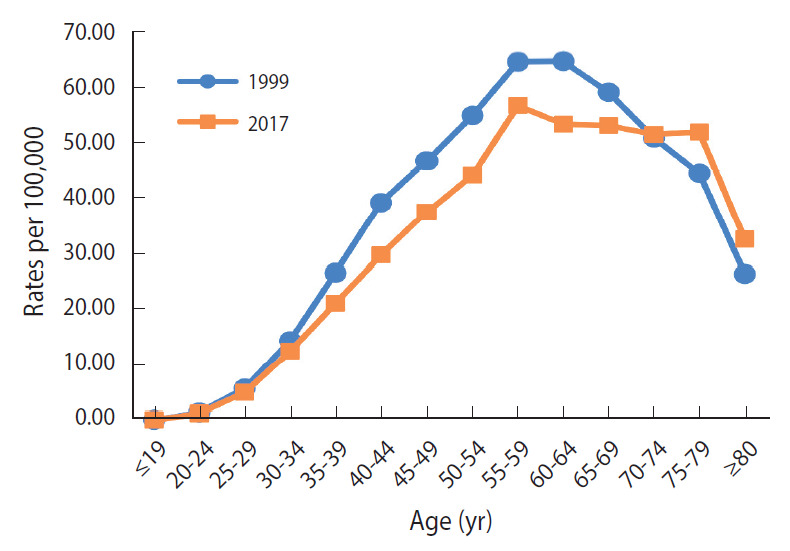
Age-specific incidence rates (per 100,000 women) for cervical cancer, Vietnam, between 1999 and 2017. Source from Institute for Health Metrics and Evaluation, Global Burden of Disease Study 2017 results. Age-adjusted by using the Vietnamese women population in 1999 and 2017.

In Vietnam, the estimated ASRs for the incidence of cervical cancer slightly declined from 21.23 (1999) to 18.12 (2017) per 100,000 (Table 1). There was a statistically significant decline in the estimated ASR for incidence, which annually decreased by 0.84% (p<0.05) (Figure 1A). As shown in Figure 2, the incidence rate in the 30-year to 69-year age group was higher in 1999 than in 2017, whereas the rate among women aged 70 years or over was higher in 2017 than in 1999. The highest ASR for incidence was consistently in the 55-year to 59-year age group in both 1999 and 2017.

**Figure 3.**
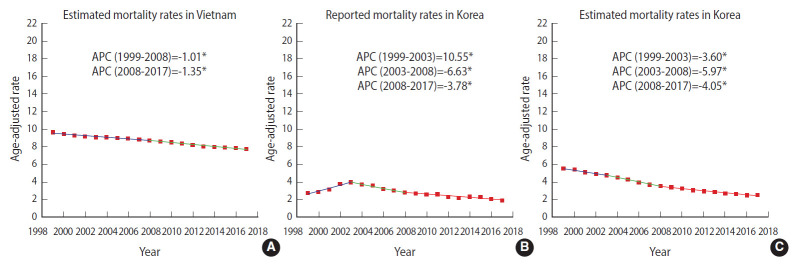
Trends in (A) estimated age-standardized mortality in Vietnam, (B) reported mortality rates in Korea, and (C) estimated age-standardized mortality in Korea for cervical cancer, 1999-2017. Age-adjusted rates: age-standardized rate (ASR) per 100,000 women. ASRs were calculated using the estimates of mortality for cervical cancer in Vietnam and Korea obtained from Global Burden of Disease Study 2017, annual population from World Population Prospects 2019; reported ASRs were calculated using mortality data, and annual population of Korea from Korea Statistical Information Service; and World Health Organizaion new world standard population. ASR and annual percent changes (APC) were computed by using Joinpoint Regression Analysis program provided by the Surveillance Research Program of the US National Cancer Institute. ^*^p<0.05.

**Figure 4.**
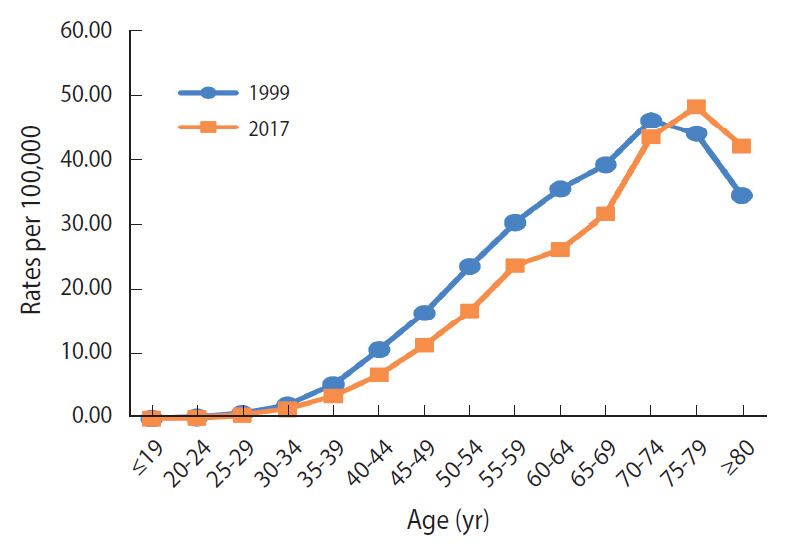
Age-specific mortality rates (per 100,000 women) for cervical cancer, Vietnam, between 1999 and 2017. Source from Institute for Health Metrics and Evaluation, Global Burden of Disease Study 2017 results. Age-adjusted by using the Vietnamese women population in 1999 and 2017.

The ASRs for estimated mortality from cervical cancer in Vietnam also gradually decreased during 1999-2017 (Table 1). As shown in Figure 3A, the estimated ASRs for mortality gradually declined until 2008 (1999-2008 APC, -1.01%; p<0.05), and then moderately decreased in subsequent years (2008-2017 APC, -1.35%; p<0.05). The ASR for mortality among women aged 30-74 years was also higher in 1999 than in 2017 (Figure 4). The highest incidence rate was in the 70-year to 74-year age group in 1999 (46.1 per 100,000), but in the 75-year to 79-year age group in 2017 (48.3 per 100,000).

In Korea, the reported incidence showed a significant declining trend (Figure 1B). The reported incidence rate substantially dropped until 2007 (1999-2007 APC, -4.53%; p<0.05), and then modestly decreased in the following years (2007-2017 APC, -2.71%; p<0.05). Although similar reduction tendencies were observed in both the reported ASR incidence and the estimated ASR incidence over time, the reported incidence rates of Korea were higher than the estimated incidence rates (Figure 1B and C). The reported ASR for mortality significantly rose until 2003 (1999-2003 APC, 10.55%; p<0.05) before falling annually by 6.63% during 2003-2008 (p<0.05), and then stably declined in the next years (2008-2017 APC, -3.78%; p<0.05) (Figure 3B). The shape of the trendline for the decrease in reported mortality was similar to that for estimated mortality since 2008 (Figure 3B and C). The estimated incidence rates were higher in Vietnam than the reported incidence rates in Korea over time (Figure 1A and B). Although a gradual decline in incidence in Vietnam was observed over time (1999-2017 APC, -0.84%), Korea experienced a marked drop in reported incidence during 1999-2007 (APC, -4.53%), followed by a steady decrease (2007-2017 APC, -2.72%). A declining trend in mortality was observed in both countries, but the estimated mortality rates were also higher in Vietnam than the reported mortality in Korea (Figure 3A and B). The estimated mortality trend in Vietnam showed a gradual decrease (1999-2008 APC, -1.01%; 2008-2017 APC, -1.35%), while reported mortality in Korea stably decreased during 2003-2008 (APC, -3.78%), then dramatically fell (2008-2017 APC, -6.63%).

## DISCUSSION

In Vietnam, cervical cancer incidence and mortality slightly decreased during 1999-2017. The APCs of incidence and mortality in Vietnam showed a slow decline. The peak ASR for incidence was in the age group of 55-59 years in both 1999 and 2017. The peak ASR for mortality was found in the age group of 70-74 years in 1999, and in the age group of 75-79 years in 2017. In Korea, the reported incidence and mortality rates significantly declined after the nationwide cancer screening for cervical cancer was initiated in 1999. However, the incidence and mortality rates in Vietnam were higher than those in Korea over this time period.

### The current situation in Vietnam

In Vietnam, trends in cervical cancer incidence and mortality have shown a slight, slow decline. Compared to trends in cervical cancer incidence in 2 cities in Vietnam during 2004-2013, the falling trend in our study is similar to that in Ho Chi Minh City (2004-2013 APC, -1.5%) and is different from that in Hanoi (2004-2013 APC, 2.8%) [4]. The incidence rate for cervical cancer reported by Vuong et al. [20] using data obtained from the GLOBOCAN database and the National Cancer Institute differed from that in our study (GBD 2017). The peak age for incidence in 2017 was in the 55-59 group, whereas older age groups had the highest rates of observed incidence in Hanoi and Ho Chi Minh City (1991-2012) [21]. The peak age of incidence in Vietnam (55-59 years) in both 1999 and 2017 was different from that in Korea (65-69 years in 1999 and ≥ 80 years in 2017, data not shown). Several studies have estimated the proportion of deaths due to cervical cancer to be < 9% of the total women cancer deaths in Vietnam (2005-2006), which is similar to that in the GLOBOCAN 2012 estimates for mortality [21]. Stability in the percentage of deaths from cervical cancer over time might reflect a gradual decreasing trend in mortality from cervical cancer in Vietnam. The mortality rate in Vietnam peaked at the age of 75-79 in 2017, unlike the peak age of mortality in Korea (≥ 80 years in 2017) [17].

The declining trends in incidence and mortality from cervical cancer in Vietnam might be due to reductions in HPV infection and human immunodeficiency virus (HIV) intervention programs. The prevalence of HPV infections stably decreased in both the south and north regions of Vietnam [21]. Moreover, the effectiveness of HIV prevention programs in Vietnam has contributed to a reduction of risky sexual behaviors, particularly in women living with HIV and sex workers [22]. HIV prevention programs are likely to be helpful for early detection of cervical cancer among high-risk groups, which might enhance the provision of effective treatment for precancerous lesions [23]. Additionally, a greater awareness of cervical cancer among Vietnamese women of reproductive age might have contributed to a slightly declining trend in incidence and mortality of cervical cancer [24]. Furthermore, improvements in the quantity of medical treatment might have supported early diagnoses and prolonged survival for cervical cancer patients in Vietnam [9].

However, incidence and mortality data are lacking in Vietnam. Nine regional cancer registries have been launched, but are not sufficient to cover the whole Vietnamese population (over 95 million individuals) living in 63 cities and provinces [9]. The 2 population-based cancer registries of Hanoi and Ho Chi Minh City cover approximately 20% of the Vietnamese population [21], and have been used in publications of the CI5 series (IARC); furthermore, the data quality of these 2 cancer registries has been evaluated [11-13]. In these publications, only MV% was assessed, whereas DCO% and the M/I ratio were not included due to the lack of mortality data in Vietnam [11-13]. Although a death registry has been maintained since 1992, information on medical cause-related death is not compulsory on death certificates [25].

In Vietnam, a nationwide population-based cancer screening for cervical cancer has not been implemented. Screening is only performed spontaneously or opportunistically, and screening tests are not covered by National Health Insurance [26]. During 2008-2016, some pilot screening projects for cervical cancer were organized with support from the National Cancer Control Program and other international organizations [9,27,28]. The coverage of these screening programs was only 2% of the target population due to the lack of follow-up schedules and financial and human resources [9,10]. Additionally, the lack of appropriate knowledge, beliefs, and behaviors among individuals regarding cancer prevention contributed to the low proportion of participants in these screening programs [9]. Although reproductiveage women have a high-level of awareness of cervical cancer, the knowledge of cervical cancer is insufficient among those living in both urban and rural areas [24]. Thus, health education related to cervical cancer should be provided in order to improve the knowledge of cervical cancer and to promote the uptake of cervical cancer screening among adult women [24,29].

Several risk factors associated with cervical cancer have been also considered for tight control measures in Vietnam. The HPV prevalence ranges from 6.1% to 8.4% among Vietnamese women and an estimated 97% of cervical cancer cases in Vietnam are attributable to HPV infection [4]. Despite the high cost of HPV vaccines, an HPV vaccination program is in the pilot process and will be included in the national immunization program in the following years [9]. For smoking, Vietnam’s efforts in terms of antitobacco actions and policies for tobacco control have contributed to stability in the prevalence of tobacco use among women (2.6-3.6% during 2002-2010) [9,21]. Regarding unhealthy diet, the prevalence of a low intake of fruits and vegetables was as high as 80% of the general population, and the consumption of salt by individuals (10-15 g daily) was 2-3 times higher than the WHO recommendation [9]. Enhancing healthy diets is one of the major priorities of cancer control strategies in Vietnam [9]. Furthermore, oral contraceptives are more commonly used, with an increase in the prevalence of their use from 11.9% (2004) to 18.8% (2015) [21]. The increasing use of oral contraceptives may hinder the decreasing trend in the incidence of cervical cancer among Vietnamese women [21]. Due to the successful family planning program, the number of births per woman decreased from 6.1 (1969-1974) to 2.1 (2015) [21]. The percentage of women having 3 or more births also declined from 20.8% (2005) to 14.2% (2012) [21].

### The National Cancer Screening Program and other prevention programs in Korea

In Korea, decreasing trends in incidence and mortality, with similar patterns, were observed in both estimated and reported rates. This comparison indicates that the estimates of incidence and mortality from the GBD 2017 can be used to monitor the burden of disease in Vietnam, which is lacking nationwide data. The reported incidence and mortality rates in Korea dropped more substantially than the estimated incidence and mortality rates in Vietnam. This might have been because the population-based screening program and other prevention programs were effective in reducing the cervical cancer burden in Korea.

In 1980, Korean Ministry of Health and Welfare established the KCCR, a national hospital-based cancer registry [6]. The KCCR data, including hospital data, 11 population-based regional cancer registries, and additional medical chart reviews, covers approximately 90% of new cases for all cancer sites [6]. In 2005, the first national incidence statistics for cancer in 1999-2002 were produced by the KCCR [17,30]. Since 2007, the KCCR has published an annual report on cancer incidence, survival, and prevalence in Korea, in which indices of data quality have been evaluated, including the M/I ratio, MV%, and DCO% [17]. The completeness of the Korean cancer registry data was reported to be as high as 98.2% in 2015 and 2017 [31]. Korea is one of a few Asian nations where cancer registry data are available to access and utilize.

The National Cancer Screening Program, including cervical cancer screening, has been offered to the low-income population since 1999 [32]. Pap smear tests are offered free-of-charge to screen for cervical cancer due to the low cost of the tests [5,33]. Guidelines for the early screening of cervical cancer in the general population developed by the Korean National Cancer Center currently recommended cervical cancer screening tests (Pap smears) for all women aged 20 and older at 2-year intervals [33]. The effectiveness of the screening program has been evaluated using cancer registry data. In addition, a free HPV vaccination program for young girls aged 12 was introduced in 2016. Moreover, health education is effective in creating awareness for and improving the knowledge of reproductive-aged women about cervical cancer and prevention, particularly in younger girls [34,35].

### Other countries that implemented organized cancer screening programs

In Vietnam, the incidence peaked in the 55-year to 59-year age group and mortality peaked in the 75-year to 79-year age group in 2017. To reduce the burden of cervical cancer among middle-aged and older women, a population-based screening program should be implemented for younger women. In several countries, a reduction of the incidence and mortality of cervical cancer was observed due to population-based cervical cancer screening using Pap smears established for younger women [36]. For instance, since Finland initiated a nationwide cervical cancer screening program for women aged from 30 to 50 in 1960s, an 80% drop of incidence and mortality rates for cervical cancer in all age groups was observed from 1965 to 2003 [37]. In Italy, which implemented population-based screening for cervical cancer in 1996, a stably decreasing trend in cervical cancer incidence was observed 17 years later, with a decline by 2.7% per year (1994-2013) [38].

Beneficial outcomes of implementing national cancer screening programs for cervical cancer have been reported in several Asian countries where the situation in terms of the health system and the cervical cancer burden was similar to that in Vietnam. In Thailand, a national cervical cancer screening program using VIA tests was organized for women aged 35-60 years at 5-year intervals in 2005, which provided good-quality evidence with a low-cost approach [39,40]. After the population-based cervical cancer screening program was launched, a steep decrease in nationwide incidence was observed in Thailand, with an annual decrease of 4.4% until 2012 [41]. In other countries where a national cervical cancer screening was available, the screening coverage was 19.9% in suburban areas and 29.1% in urban areas in China 1 year after the implementation of the program (2009), and 7.3% of the target population in Indonesia 5 years after launching the program (2014) [40]. Although the coverage of screening in China and Indonesia is low, the implementation of these programs might reduce the burden of cervical cancer in following years [40].

### Strengths and limitations

Based on estimated national data for incidence and mortality from the GBD 2017 study, we overviewed the burden of cervical cancer in Vietnam under the circumstances of lacking data, instead of only providing data from large cities or provinces, as in previous studies [20]. Furthermore, we also provided evidence of the achievements of Korea, where the National Cancer Screening Program has been implemented for the targeted at-risk population, with a population-based central cancer registry [6,32].

A limitation of our study is that we used secondary data from the GBD 2017 with estimates of incidence and mortality for cervical cancer in Vietnam that were gathered from various sources, such as cancer registry data, previous studies, surveillance data, and others [15]. The estimates from the GBD 2017 study are known to depend on the sources for each country, but it remains the best choice to overview the cervical cancer burden under current circumstances in Vietnam. Furthermore, we provided both estimated and reported data for the incidence and mortality of cervical cancer in Korea, which could not be done in Vietnam due to the lack of national data. In the comparison between estimated and reported rates in Korea, a similar pattern of trends was reported for both reported and estimated incidence and mortality, but the reported incidence of cervical cancer was higher than the estimated incidence. This implies that the current actual incidence of cervical cancer in Vietnam might be higher than the estimated incidence rate. Thus, a population-based cancer registry should be established to accurately evaluate the nationwide burden of cervical cancer in Vietnam and also to monitor the activities of a national cancer screening program.

In conclusion, the ASRs for the incidence and mortality of cervical cancer in Vietnam slightly declined from 1999 to 2017, whereas the reported incidence and mortality of cervical cancer in Korea rapidly declined since the National Cancer Screening Program was implemented. Under the circumstances in Vietnam, the introduction of a national cancer screening program for Vietnamese women aged 30 years and older may maintain a steadily decreasing trend in the incidence and mortality of cervical cancer. Furthermore, this study also implies that it is necessary to develop a nationwide cancer registry, and that enhancing its coverage could help monitor the effectiveness of the program.

## Figures and Tables

**Table 1.** Counts, crude rates, and age-standardized rates (ASRs) for the estimated incidence and mortality of cervical cancer annually in Vietnam, 1999-2017^1^

Year	Incidence			Mortality		
	Cases	Crude (per 100,000)	ASR (per 100,000)^2^	Cases	Crude (per 100,000)	ASR (per 100,000)^2^
1999	6,559	16.44	21.23	2,964	7.43	9.64
2000	6,643	16.47	20.82	2,993	7.42	9.44
2001	6,752	16.57	20.52	3,037	7.45	9.29
2002	6,874	16.70	20.29	3,087	7.50	9.18
2003	7,031	16.92	20.19	3,152	7.59	9.12
2004	7,204	17.18	20.10	3,225	7.69	9.06
2005	7,369	17.42	19.93	3,292	7.78	8.96
2006	7,554	17.70	19.90	3,368	7.89	8.93
2007	7,710	17.91	19.74	3,430	7.97	8.83
2008	7,861	18.10	19.54	3,487	8.03	8.71
2009	8,047	18.37	19.41	3,556	8.12	8.62
2010	8,230	18.61	19.25	3,625	8.20	8.51
2011	8,318	18.63	18.95	3,647	8.17	8.34
2012	8,447	18.73	18.72	3,686	8.17	8.20
2013	8,601	18.88	18.54	3,738	8.20	8.08
2014	8,785	19.09	18.41	3,805	8.27	7.99
2015	8,982	19.32	18.29	3,876	8.34	7.88
2016	9,187	19.57	18.22	3,955	8.43	7.83
2017	9,375	19.77	18.12	4,034	8.51	7.76

1 From the Institute for Health Metrics and Evaluation, Global Burden of Disease Study 2017 results.

2 ASRs were calculated using the Vietnamese women population (1999-2017) from World Population Prospects 2019 Project and then adjusted using the World Health Organization standard population.
